# Investigating age-stratified outcomes following surgical fixation of humeral shaft fractures in the elderly

**DOI:** 10.1007/s00068-025-02992-7

**Published:** 2025-10-28

**Authors:** Maxwell Ruffner, Olivia Stala, Jared Sasaki, Victor Koltenyuk, Jack W. Weick

**Affiliations:** 1 New York Medical College, Valhalla, USA; 2 Department of Orthopedic Trauma and Reconstructive Surgery, Grant Medical Center, Columbus, USA

**Keywords:** Orthopaedics, Geriatrics, Humeral shaft fracture, Trauma

## Abstract

**Purpose:**

As the global population ages, fractures among elderly individuals are increasing, with humeral shaft fractures (HSFs) comprising 3% of long bone fractures. While chronological age has been shown to influence surgical outcomes in elderly patients, there is paucity of literature examining age-specific differences in outcomes for HSF management. Given variations in bone quality, comorbid conditions, and functional status among elderly patients, an age-stratified approach may be necessary. This study aims to compare in-hospital outcomes following HSF fixation across geriatric age groups to inform treatment strategies.

**Methods:**

The National Inpatient Sample database (2015–2021) was queried for patients with HSFs and subsequent fixation. Patients were stratified into three groups: <65, 65–79, and ≥ 80 years. Multivariate logistic regression assessed associations between each age group and adverse outcomes. A secondary analysis was done comparing patients ≥ 80 with those aged 65–79.

**Results:**

A total of 5,276 patients were included in the three-group analysis. Both the 65–79 and ≥ 80 cohorts had higher odds of extended length of stay (eLOS), non-home discharge (NHD), and acute kidney injury (AKI). Only the ≥ 80 group was associated with increased odds of in-hospital mortality. After excluding those < 65, 2,777 patients remained for the two-group analysis. The ≥ 80 group was associated with increased odds of mortality, eLOS, and NHD compared to patients aged 65–79.

**Conclusion:**

Our study underscores the importance of considering an age-stratified approach to surgical management of HSFs in the elderly. These results provide further information for clinicians to help guide patients and family in clinical decision making for geriatric HSFs.

**Supplementary Information:**

The online version contains supplementary material available at 10.1007/s00068-025-02992-7.

## Introduction

 The global population is aging rapidly, with a significant increase in the proportion of individuals aged 70 years and older [1, 2]. This demographic shift has led to a rise in the incidence of fractures, particularly among the elderly, due to factors such as osteoporosis, increased fall risk, and reduced physical resilience. Humerus fractures are a common injury in the elderly population, with humeral shaft fractures (HSFs) representing around 3% of all long bone fractures [3]. The management of such fractures often involves a combination of surgical and non-surgical approaches, with the choice of treatment influenced by factors such as fracture severity, degree of displacement, nonunion, patient comorbidities, and functional status [4].

Elderly patients may have different postoperative outcomes based on marked differences between age brackets, including physiological changes, changes in bone density, differences in health status, and altered functional independence [5]. These differences are important to understand to optimize perioperative care, accurately stratify risk, and appropriately counsel patients.

Previous studies have shown that chronological age can influence outcomes in elderly patients undergoing orthopedic surgeries. For instance, Magnuson et al. reported higher rates of complications and mortality in octogenarians undergoing certain orthopedic procedures, such as revision total hip arthroplasty [6]. However, other studies have reported that septuagenarians and octogenarians with proximal humerus fractures had comparable outcomes to younger geriatric patients, suggesting that age alone should not be a contraindication for surgical intervention [7]. Despite the growing body of literature, these findings highlight the paucity in work specifically comparing HSF management outcomes and distinguishing between these subgroups of older age. This gap in knowledge is particularly relevant for HSFs, where the optimal treatment approach may vary significantly across these age groups due to differences in bone quality, comorbid conditions, and functional status.

The current study seeks to fill this gap by comparing in-hospital outcomes for HSFs in elderly patients, with the goal of guiding surgical decision-making as well as improving outcomes for these growing populations.

## Methods

### Database characteristics

We examined National Inpatient Sample (NIS) data from 2015 to 2021. It is a publicly available database developed for the Healthcare Cost and Utilization Project (HCUP) containing data from over 7 million hospital stays annually [8]. The NIS constitutes the largest all-payer inpatient database in the United States, approximating a 20% sample of the nation’s hospitalizations. It is drawn from all states, covering over 97% of the US population.

### Compliance with ethical guidelines

The NIS is a de-identified database that does not directly involve human subjects. All facets of the HCUP Nationwide Database Data Use Agreement were upheld.

### Patient selection

Adult patients 18 years of age or older with ICD-10-PCS code for HSF fixation (ICD-10 PCS codes beginning with 0PSF, 0PSG). Patients presenting for subsequent encounters or sequelae for such fractures were excluded, as well as those also presenting with fractures in other parts of the body. Patients with open fractures were included in the study and defined as a variable that was controlled for in multivariable analysis. All ICD-10 codes used for inclusion and exclusion criteria are enumerated in Online Resource 1. Patient characteristics included age group (< 65, 65–79, and ≥ 80), sex, APR-DRG score, and open vs. closed fracture type.

### Outcomes

The primary outcomes were in-hospital patient mortality, extended length of stay (eLOS), and non-home discharge (NHD). eLOS was defined as LOS greater than the median. Other outcomes of interest were deep vein thrombosis (DVT), acute kidney injury (AKI), pulmonary embolism (PE), myocardial infarction (MI), pneumonia, infection, and sepsis. All outcomes refer to in-hospital events due to the nature of the NIS database.

### Statistical analysis

Patient characteristics were displayed as frequencies (%) for categorical variables and medians [IQR] for continuous variables. Multivariable logistic regression was performed to examine the odds of in-hospital complications for each age group, controlling for sex, APR-DRG score, and open vs. closed fracture. A subgroup analysis was done with only those patients 65 years of age or older, comparing the same outcomes in the 65–79 years group to the over 80 group. The same variables: sex, APR-DRG score, and open vs. closed fracture were controlled for in this model as well. Statistical significance was set at *p* < 0.05. All analyses were performed using the python programming language.

## Results

### Patient demographics and outcomes

This study included 5,276 patients that underwent surgery for HSF. Of these patients, 2,499 (47.3%) were less than 65 years of age, 1,728 (32.7%) were between ages 65 and 79, and 1,049 (19.9%) were older than 80 years. Baseline patient demographics and comorbidities are shown in Table 1. Of note, most patients in this population were White (72.4%) and female (63.3%). The most common comorbidity was uncomplicated hypertension (42.1%). The most common 30-day postoperative complications were AKI (9.9%), pneumonia (1.8%), and sepsis (1.1%). Other postoperative complications included in-hospital mortality (0.6%) and DVT (0.7%).

**Table 1:** Univariate analysis of patient demographics, comorbidities, and in-hospital complications for elderly patients who underwent HSF fixation across three age groups

	All (*n* = 5276)	< 65 years (*n* = 2499)	65–79 years (*n* = 1728)	>= 80 years (*n* = 1049)	*p*-value
Female Sex	3344 (63.38%)	1269 (50.78%)	1274 (73.73%)	801 (76.36%)	< 0.001
Race					< 0.001
White	3822 (72.44%)	1477 (59.10%)	1446 (83.68%)	899 (85.70%)	
Black	545 (10.33%)	447 (17.89%)	74 (4.28%)	24 (2.29%)	
Hispanic	516 (9.78%)	336 (13.45%)	107 (6.19%)	73 (6.96%)	
Asian/Pacific Islander	75 (1.42%)	44 (1.76%)	19 (1.10%)	12 (1.14%)	
Native American	32 (0.61%)	26 (1.04%)	6 (0.35%)	0	
Other	138 (2.62%)	94 (3.76%)	30 (1.74%)	14 (1.33%)	
Comorbidities					
AIDS	18 (0.34%)	11 (0.44%)	7 (0.41%)	0	0.100
Alcohol abuse	434 (8.23%)	300 (12.00%)	127 (7.35%)	7 (0.67%)	< 0.001
Autoimmune	185 (3.51%)	64 (2.56%)	80 (4.63%)	41 (3.91%)	0.001
Lymphoma	26 (0.49%)	7 (0.28%)	10 (0.58%)	9 (0.86%)	0.067
Leukemia	11 (0.21%)	1 (0.04%)	5 (0.29%)	5 (0.48%)	0.023
Metastasis	78 (1.48%)	19 (0.76%)	40 (2.31%)	19 (1.81%)	< 0.001
Solid Cancer	65 (1.23%)	9 (0.36%)	31 (1.79%)	25 (2.38%)	< 0.001
Dementia	394 (7.47%)	21 (0.84%)	133 (7.70%)	240 (22.88%)	< 0.001
Depression	862 (16.34%)	319 (12.77%)	370 (21.41%)	173 (16.49%)	< 0.001
Diabetes, Uncomplicated	672 (12.74%)	231 (9.24%)	289 (16.72%)	152 (14.49%)	< 0.001
Diabetes, Complicated	667 (12.64%)	177 (7.08%)	347 (20.08%)	143 (13.63%)	< 0.001
Drug Abuse	159 (3.01%)	124 (4.96%)	31 (1.79%)	4 (0.38%)	< 0.001
Hypertension, uncomplicated	821 (15.56%)	129 (5.16%)	367 (21.24%)	325 (30.98%)	< 0.001
Hypertension, complicated	2223 (42.13%)	730 (29.21%)	960 (55.56%)	533 (50.81%)	< 0.001
Lung Chronic	942 (17.85%)	348 (13.93%)	394 (22.80%)	200 (19.07%)	< 0.001
Obesity	1075 (20.38%)	507 (20.29%)	457 (26.45%)	111 (10.58%)	< 0.001
Perivascular	166 (3.15%)	37 (1.48%)	73 (4.22%)	56 (5.34%)	< 0.001
Hypothyroidism	840 (15.92%)	201 (8.04%)	360 (20.83%)	279 (26.60%)	< 0.001
Thyroid other	46 (0.87%)	16 (0.64%)	19 (1.10%)	11 (1.05%)	0.227
APRDRG Risk Mortality					< 0.001
Minor	176 (3.34%)	58 (2.32%)	69 (3.99%)	49 (4.67%)	
Moderate	589 (11.16%)	130 (5.20%)	255 (14.76%)	204 (19.45%)	
Major	3214 (60.92%)	1967 (78.71%)	881 (50.98%)	366 (34.89%)	
Extreme	1297 (24.58%)	344 (13.77%)	523 (30.27%)	430 (40.99%)	
Open Fracture	558 (10.58%)	414 (16.57%)	91 (5.27%)	53 (5.05%)	< 0.001
Complications					
Mortality	34 (0.64%)	6 (0.24%)	11 (0.64%)	17 (1.62%)	< 0.001
LOS	3 [2–5]	2 [2–4]	3 [2–5]	4 [3–6]	< 0.001
Extended LOS	2231 (42.29%)	806 (32.25%)	808 (46.76%)	617 (58.82%)	< 0.001
Non-home discharge	1734 (32.87%)	282 (11.28%)	728 (42.13%)	724 (69.02%)	< 0.001
Deep Vein Thrombosis	35 (0.66%)	12 (0.48%)	13 (0.75%)	10 (0.95%)	0.244
Acute Kidney Injury	523 (9.91%)	130 (5.20%)	214 (12.38%)	179 (17.06%)	< 0.001
Pulmonary Embolism	18 (0.34%)	2 (0.08%)	10 (0.58%)	6 (0.57%)	0.009
Myocardial Infarction	31 (0.59%)	7 (0.28%)	15 (0.87%)	9 (0.86%)	0.021
Pneumonia	97 (1.84%)	28 (1.12%)	44 (2.55%)	25 (2.38%)	0.001
Infection	12 (0.23%)	8 (0.32%)	2 (0.12%)	2 (0.19%)	0.376
Sepsis	58 (1.10%)	18 (0.72%)	21 (1.22%)	19 (1.81%)	0.015

Categorical variables are presented as counts with percentages and continuous variables are presented as medians with interquartile ranges

### Multivariable analysis

A multivariable regression analysis was done to compare in-hospital outcomes of patients under the age of 65 to patients aged between 65 and 79 and patients aged over the age of 80. This regression controlled for sex, open vs. closed fracture and APRDRG Risk Mortality. Results showed that patients aged 65–79 were at significantly increased odds of eLOS (OR: 1.29, 95% CI: [1.12–1.49] *p* = 0.001), NHD (OR: 4.29, 95% CI [3.63–5.07] *p* < 0.001), and AKI (OR: 1.37, 95% CI: [1.04–1.81] *p* = 0.027). Patients over the age of 80 were at significantly increased risk of in-hospital mortality (OR: 6.36, 95% CI: [1.93, 20.92] *p* = 0.002), eLOS (OR: 1.77, 95% CI: [1.49–2.10] *p* < 0.001), NHD (OR: 12.16, 95% CI: [10.04–14.73] *p* < 0.001), and AKI (OR: 1.64, 95% CI: [1.23–2.20] *p* = 0.001) (Table 2).

**Table 2:** Multivariable logistic regression for in-hospital complications controlling for sex, open vs. closed fracture, and APRDRG risk of mortality

Age Group (ref*: <65)	65–79		≥ 80	
Outcome	OR [95% CI]	*p*-value	OR [95% CI]	*p*-value
Mortality	2.39 [0.69, 8.21]	0.168	**6.36 [1.93**,** 20.92]**	**0.002**
Extended LOS	**1.29 [1.12**,** 1.49]**	**0.001**	**1.77 [1.49**,** 2.10]**	**< 0.001**
Non-home Discharge	**4.289 [3.63**,** 5.07]**	**< 0.001**	**12.16 [10.04**,** 14.73]**	**< 0.001**
DVT	1.40 [0.60, 3.31]	0.438	1.52 [0.61, 3.81]	0.370
AKI	**1.37 [1.04**,** 1.81]**	**0.027**	**1.64 [1.23**,** 2.20]**	**0.001**
PE	4.72 [0.97, 22.89]	0.054	3.81 [0.72, 20.10]	0.115
MI	1.66 [0.64, 4.29]	0.298	1.32 [0.46, 3.74]	0.602
Pneumonia	1.00 [0.59, 1.69]	0.99	0.72 [0.40, 1.31]	0.285
Infection	0.26 [0.05, 1.30]	0.101	0.38 [0.07, 1.92]	0.240
Sepsis	0.80 [0.40, 1.60]	0.523	1.00 [0.486, 2.05]	0.994

*ref = reference category

An additional multivariate regression analysis was conducted, restricted to patients aged 65 and older. After excluding those younger than 65, outcomes were compared between the 65–79 and ≥ 80 age groups, controlling for sex, fracture type (open vs. closed), and APR-DRG risk of mortality. The results showed that compared to those aged 65–79, age over 80 is associated with significantly higher odds of in-hospital mortality (OR: 2.66 [95% CI: 1.15–6.18] *p* = 0.023), eLOS (OR: 1.39 [95% CI: 1.18–1.64] *p* < 0.001), and NHD (OR: 2.83 [95% CI: 2.39–3.35] *p* < 0.001) (Table 3).

**Table 3:** Multivariable logistic regression for in-hospital outcomes of patients aged 65 and older controlling for sex, fracture type (open vs. closed) and APRDRG risk of mortality

Outcome	OR [95% CI]	*p*-value
Mortality		
Sex (ref*: male)	0.59 [0.26, 1.34]	0.205
APRDRG Risk Mortality	**13.19 [6.56**,** 26.52]**	**< 0.001**
Age 80 (ref*: 65–80)	**2.66 [1.15**,** 6.18]**	**0.023**
Fracture Type (ref*: closed)	1.88 [0.56, 6.35]	0.308
Extended LOS		
Sex (ref*: male)	1.11 [0.92, 1.33]	0.291
APRDRG Risk Mortality	**2.60 [2.34**,** 2.90]**	**< 0.001**
Age 80 (ref*: 65–80)	**1.39 [1.18**,** 1.64]**	**< 0.001**
Fracture Type (ref*: closed)	1.18 [0.82, 1.72]	0.375
Non-home Discharge		
Sex (ref*: male)	1.09 [0.90, 1.31]	0.377
APRDRG Risk Mortality	**2.08 [1.88**,** 2.30]**	**< 0.001**
Age 80 (ref*: 65–80)	**2.83 [2.39**,** 3.35]**	**< 0.001**
Fracture Type (ref*: closed)	0.85 [0.59, 1.24]	0.402
Deep Vein Thrombosis		
Sex (ref*: male)	1.34 [0.51, 3.50]	0.551
APRDRG Risk Mortality	**2.84 [1.84**,** 4.39]**	**< 0.001**
Age 80 (ref*: 65–80)	1.07 [0.46, 2.47]	0.879
Fracture Type (ref*: closed)	**5.38 [2.03**,** 14.25]**	**0.001**
Acute Kidney Injury		
Sex (ref*: male)	0.81 [0.62, 1.07]	0.141
APRDRG Risk Mortality	**5.76 [4.88**,** 6.78]**	**< 0.001**
Age 80 (ref*: 65–80)	1.20 [0.93, 1.54]	0.17
Fracture Type (ref*: closed)	0.681 [0.382, 1.212]	0.191
Pulmonary Embolism		
Sex (ref*: male)	0.87 [0.30, 2.46]	0.789
APRDRG Risk Mortality	**5.03 [2.75**,** 9.17]**	**< 0.001**
Age 80 (ref*: 65–80)	0.81 [0.29, 2.28]	0.694
Fracture Type (ref*: closed)	2.78 [0.74, 10.43]	0.13
Myocardial Infarction		
Sex (ref*: male)	0.72 [0.31, 1.67]	0.441
APRDRG Risk Mortality	**5.92 [3.51**,** 9.98]**	**< 0.001**
Age 80 (ref*: 65–80)	0.80 [0.34, 1.88]	0.609
Fracture Type (ref*: closed)	1.59 [0.44, 5.78]	0.479
Pneumonia		
Sex (ref*: male)	1.22 [0.70, 2.14]	0.485
APRDRG Risk Mortality	**5.27 [3.91**,** 7.10]**	**< 0.001**
Age 80 (ref*: 65–80)	0.72 [0.43, 1.22]	0.226
Fracture Type (ref*: closed)	0.15 [0.02, 1.11]	0.063
Infection		
Sex (ref*: male)	n/a	0.999
APRDRG Risk Mortality	2.23 [0.81, 6.17]	0.121
Age 80 (ref*: 65–80)	1.39 [0.20, 9.98]	0.741
Fracture Type (ref*: closed)	5.55 [0.56, 54.93]	0.143
Sepsis		
Sex (ref*: male)	1.22 [0.59, 2.53]	0.587
APRDRG Risk Mortality	**10.76 [6.42**,** 18.04]**	**< 0.001**
Age 80 (ref*: 65–80)	1.30 [0.66, 2.55]	0.454
Fracture Type (ref*: closed)	2.12 [0.78, 5.76]	0.143

*ref = reference category

## Discussion

This study aimed to evaluate the differential impact of advanced age on in-hospital outcomes following surgical fixation of HSFs. Our findings revealed a clear gradient of risk. While both elderly groups (65–79 and ≥ 80) had increased odds of eLOS, NHD, and AKI compared to those under 65, only the ≥ 80 group had significantly higher in-hospital mortality. In the elderly-only analysis, patients aged 80 and older had more than double the risk of death, as well as significantly increased risks of eLOS and NHD. These results emphasize the need for nuanced perioperative management in adults of advanced age undergoing orthopedic trauma surgery.

These findings align with and help contextualize previous research highlighting that advanced age is a key risk factor for adverse perioperative outcomes in orthopedic trauma. In our study, patients with HSFs aged 80 and older faced significantly higher odds of in-hospital mortality, extended hospitalization, and non-home discharge compared to both younger elderly patients and those under 65. These trends are consistent with data from other fracture types, such as ankle and tibial plateau fractures, where octogenarians experienced elevated risks of in-hospital mortality, sepsis, renal complications, and discharge to facilities compared to septuagenarians [9, 10]. Similarly, the increased risk of complications we observed among the oldest patients mirrors findings from arthroplasty literature, where nonagenarians undergoing shoulder or hip procedures demonstrated sharply increased complication rates relative to younger cohorts [6, 11]. These studies support the notion that beyond a certain physiologic threshold, specifically ages of 80 and above, older adults may be less able to tolerate the physiological stress of surgery, particularly in the acute trauma setting. Our findings apply this understanding specifically to HSFs, which are relatively underexplored in this context, and suggest that age-related physiologic vulnerability may compound the challenges of urgent fracture fixation in this population.

Our results also suggest that age may serve as a proxy for a broader set of underlying risk factors such as frailty, multiple comorbidities, and reduced physiological reserve, which collectively contribute to poorer outcomes. For example, the increased odds of AKI we observed in both elderly groups may reflect decreased baseline renal function and higher rates of dehydration or medication use in older adults. Several prior studies support this interpretation, showing that in various surgical contexts, factors like high ASA scores, poor nutritional status, diabetes, and urgency of surgery are often stronger predictors of complications than age alone [12–14]. This suggests that the elevated in-hospital mortality and non-home discharge we found in octogenarians may not be due solely to chronological age, but rather to the clustering of age-associated risks that are especially pronounced in trauma settings. Moreover, population-level data indicate that adverse outcomes increase steeply beginning around ages 85–90, which aligns with the accelerated risk we observed in our ≥ 80 cohort [15]. Taken together, these patterns reinforce the idea that while age is a useful stratification tool, it is ultimately the interplay between age, physiological reserve, and surgical stress that determines outcomes.

Importantly, these findings are not limited to high-acuity or emergent procedures. Elective orthopedic surgeries, which typically allow more time for surgical planning and patient optimization, also demonstrate clear age-related disparities in outcomes. For example, it was found that nonagenarians undergoing total joint arthroplasty experienced significantly higher in-hospital complication rates and longer lengths of stay compared to carefully matched octogenarians, despite similar 90-day outcomes [16]. Similarly, it was reported that while robotic-assisted surgery for endometrial cancer was generally feasible in patients over 80, postoperative complication rates remained notably higher in this age group compared to younger cohorts [17]. These procedures, performed in elective settings, benefit from the opportunity to optimize comorbidities, perform thorough risk assessments, and engage in multidisciplinary preoperative planning. In contrast, trauma surgeries like those for humeral shaft or tibial plateau fractures are often urgent, leaving limited time for such optimization. As a result, the impact of advanced age on outcomes may be even more pronounced in trauma contexts, where physiologic reserve is acutely tested and time-sensitive decision-making is critical.

These findings suggest that while age ≥ 80 should not serve as an automatic exclusion criterion for surgical intervention, it is undeniably associated with increased short-term risks such as in-hospital mortality, extended hospital stay, and discharge to non-home settings. This reinforces the critical importance of thorough preoperative planning and individualized care in this age group. Rather than using age as a blunt cutoff, clinicians should engage in detailed risk-benefit discussions that incorporate comorbidities, functional status, and patient goals. Proactive strategies such as preoperative medical optimization, early involvement of geriatric teams, and realistic discharge planning, can help reduce complication rates and improve recovery trajectories for very elderly patients [18]. Moreover, our results emphasize the need to differentiate between elderly subgroups, as those aged 80 and above represent a rapidly growing population with unique physiological vulnerabilities and healthcare needs. Recognizing and responding to these differences with tailored care pathways and risk stratification models will be essential as life expectancy rises and more octogenarians and nonagenarians present for surgical care [19].

### Limitations

Our study has several limitations that should be acknowledged. First, the NIS database is limited to single episodes of acute care and lacks follow up data and does not provide post-discharge follow-up data. As a result, we were unable to assess long-term outcomes such as functional recovery, readmission rates, or post-acute rehabilitation success.

Second, although we controlled for important aspects of baseline patient risk and injury severity, these variables cannot capture the full complexity of surgical care. Sex differences have been consistently linked to variation in bone health, fracture risk, and recovery trajectories in orthopedic populations. Open versus closed fracture status is a critical determinant of infection risk, soft tissue injury, and perioperative complexity. The APR-DRG Risk of Mortality score has been validated as a measure of inpatient severity and mortality risk and is widely used in administrative database research, making it a reasonable proxy for comorbidity burden and physiologic status [20]. Together, these covariates provide a reasonable foundation for risk adjustment at the population level and support the validity of our adjusted associations. However, the NIS lacks key operative details, including operative time, fracture pattern, fixation method, intraoperative complications, and anesthesia type. These factors are well established as strong predictors of outcomes after fracture surgery. For example, randomized control trials and meta-analyses have demonstrated that anesthesia type can significantly influence short-term mortality and complication rates after hip fracture surgery [21, 22], while single-center studies incorporating fracture pattern and fixation construct show substantial variations in outcomes related to surgical technique [23]. Compared with these unmeasured operative variables, our covariates primarily address patient-level and injury-level risk rather than surgical decision-making and perioperative management. Consequently, while our models are sufficiently adjusted to identify robust population-level associations, they cannot fully account for confounding from surgical and anesthetic factors.

Third, although we attempted to adjust for comorbidity burden using the APR-DRG Risk of Mortality score, this measure may not fully account for the complexity of elderly patients’ health status. Fourth, as with any retrospective study using administrative data, there is a risk of coding errors, misclassification, and incomplete data capture, which could introduce bias into the analysis. Fifth, the NIS does not capture prehospital living arrangements. Therefore, the “non-home discharge” outcome may overestimate transitions to skilled nursing or rehabilitation facilities, particularly among patients who ordinarily reside in such settings.

Lastly, our findings reflect the sampled hospitalizations in the NIS but may not be fully generalizable to all U.S. hospitalizations. Certain hospital types (e.g. federal, rehabilitation, psychiatric hospitals) are not included in the NIS, potentially introducing bias. Nonetheless, since the NIS contains data from a large and diverse set of hospitals across U.S. census regions, our study provides valuable insight into age-stratified outcomes following HSF fixation, although exact population estimates should be interpreted with caution.

### Future directions

Future studies should aim to provide a more nuanced understanding of how frailty, functional status, and comorbidities influence surgical outcomes in elderly patients with HSFs. Prospective, multicenter studies incorporating standardized frailty assessments, functional measures, and long-term follow-up will be critical to validating and expanding upon our findings. Additionally, stratifying outcomes by surgical approach, implant type, and perioperative optimization strategies could elucidate best practices for treating this vulnerable population. Furthermore, more research is needed regarding outcomes of elderly patients who underwent operative vs. nonoperative treatment for HSF.

It would also be beneficial to explore patient-centered outcomes, such as return to baseline function, quality of life, and satisfaction with care. As the population of octogenarians and nonagenarians grows, there is a pressing need for comprehensive, individualized care pathways that account for the complex interplay of age, frailty, and surgical risk.

## Conclusion

Our study underscores the importance of nuanced, age-stratified approaches to surgical care in the elderly, particularly for patients aged 80 and older undergoing orthopedic trauma procedures. The increased risks observed in this group, particularly regarding in-hospital mortality, prolonged hospitalization, and non-home discharge, highlight the need for comprehensive perioperative strategies that go beyond chronological age alone. Our findings, supported by a growing body of literature, suggest that incorporating assessments of frailty, comorbid burden, and surgical urgency into clinical decision-making can more accurately guide treatment plans and improve outcomes. As the population continues to age and the number of octogenarians and nonagenarians requiring surgical intervention rises, the development of evidence-based, individualized care pathways will be critical to ensuring safe and effective treatment for this vulnerable demographic.

## Supplementary Information

Below is the link to the electronic supplementary material.

Supplementary Material 1

## Data Availability

No datasets were generated or analysed during the current study.
