# Supplementary material for: Investigating age-stratified outcomes following surgical fixation of humeral shaft fractures in the elderly
Source: Eur J Trauma Emerg Surg. 2025 Oct 28;51(1):307. doi: 10.1007/s00068-025-02992-7 (PMC12568814; doi:10.1007/s00068-025-02992-7)
Supplement: Supplementary file 1 — Supplementary Material 1 [file 68_2025_2992_MOESM1_ESM.docx]

**Supplementary Table 1. Patient selection and exclusion codes**

| **Diagnosis/Procedure** | **ICD-10 Code** |
| --- | --- |
| Insertion of device into or repositioning of humeral shaft | 0PSF*, 0PSG*, 0PHF*, 0PHG* |
| Initial encounter for closed fracture of shaft of humerus | S423XXA |
| Initial encounter for open fracture of shaft of humerus | S423XXB |
| Subsequent encounter or sequela for humeral shaft fracture | S423XXD, S423XXG, S423XXK, S423XXP, S423XXS |

*All ICD-10 code sub-characters under the diagnosis

**Supplementary Table 2. Patient outcome codes**

| **Diagnosis** | **ICD-10 Code** |
| --- | --- |
| Deep Venous Thrombosis | I824*, I826* |
| Acute Kidney Injury | N17* |
| Pulmonary Embolism | I26* |
| Myocardial Infarction | I21* |
| Pneumonia | J13*, J14*, J15*, J16*, J17*, J18* |
| Infection Post Procedure | T814* |
| Sepsis | A419* |

*All ICD-10 code sub-characters under the diagnosis
